# Renal tubular calcium phosphate microcrystallopathy and age-related kidney function decline: The Aging Kidney Study

**DOI:** 10.1093/ckj/sfag085

**Published:** 2026-03-16

**Authors:** Keisei Kosaki, Shoya Mori, Masahiro Matsui, Masaki Yoshioka, Jiyeon Park, Natsumi Nishitani, Shun Yoshikoshi, Wataru Murasaki, Chie Saito, Masahiko Gosho, Seiji Maeda, Makoto Kuro-o, Kunihiro Yamagata

**Affiliations:** Division of Internal Sports Medicine, Institute of Health and Sport Sciences, University of Tsukuba, Ibaraki, Japan; Advanced Research Initiative for Human High Performance, Institute of Health and Sport Sciences, University of Tsukuba, Ibaraki, Japan; Division of Preventive and Sports Nephrology, Graduate School of Comprehensive Human Sciences, University of Tsukuba, Ibaraki, Japan; Department of Nutritional Science, Faculty of Applied Bioscience, Tokyo University of Agriculture, Tokyo, Japan; Department of Cardiology, Institute of Medicine, University of Tsukuba, Ibaraki, Japan; Department of Cardiology, Institute of Medicine, University of Tsukuba, Ibaraki, Japan; Department of Child Psychiatry, Yokohama City University Hospital, Kanagawa, Japan; Division of Mineral Metabolism, Center for Molecular Medicine, Jichi Medical University, Tochigi, Japan; Japan Agency for Gerontological Evaluation Study, Chiba, Japan; Physical Fitness Research Institute, Meiji Yasuda Life Foundation of Health and Welfare, Tokyo, Japan; Division of Internal Sports Medicine, Institute of Health and Sport Sciences, University of Tsukuba, Ibaraki, Japan; Division of Preventive and Sports Nephrology, Graduate School of Comprehensive Human Sciences, University of Tsukuba, Ibaraki, Japan; Graduate School of Comprehensive Human Sciences, University of Tsukuba, Ibaraki, Japan; Department of Nephrology, Institute of Medicine, University of Tsukuba, Ibaraki, Japan; Department of Biostatistics, Institute of Medicine, University of Tsukuba, Ibaraki, Japan; Faculty of Sport Sciences, Waseda University, Saitama, Japan; Division of Preventive and Sports Nephrology, Graduate School of Comprehensive Human Sciences, University of Tsukuba, Ibaraki, Japan; Division of Mineral Metabolism, Center for Molecular Medicine, Jichi Medical University, Tochigi, Japan; Division of Preventive and Sports Nephrology, Graduate School of Comprehensive Human Sciences, University of Tsukuba, Ibaraki, Japan; Department of Nephrology, Institute of Medicine, University of Tsukuba, Ibaraki, Japan

**Keywords:** aging kidney, calcium phosphate microcrystals, estimated glomerular filtration rate slope, estimated phosphate concentration in proximal tubular fluid, serum fibroblast growth factor 23

## Abstract

**Background:**

Elevated phosphate concentration in proximal tubular fluid promotes calcium phosphate microcrystallopathy, thereby accelerating the progression of chronic kidney disease (CKD). However, the clinical significance of proximal tubular phosphate exposure in humans remains uncertain. We aimed to determine whether estimated proximal tubular fluid phosphate concentration (ePTFp) is independently associated with age-related kidney function decline in adults with and without CKD.

**Methods:**

We conducted a 5-year prospective cohort study involving 308 adults with and without CKD. ePTFp—a novel, noninvasive index—and serum fibroblast growth factor 23 (FGF23) concentrations were derived from blood and urine measurements. Kidney function decline, expressed as estimated glomerular filtration rate (eGFR) slope, was modeled using linear mixed-effects analysis. Associations of ePTFp and serum FGF23 with eGFR slope were examined using multivariable regression analysis, adjusting for potential covariates at baseline, including age, sex, several comorbidities, current smoking status, eGFR, and urinary glomerular and tubular injury markers.

**Results:**

Over 5 years, eGFR declined in participants with and without CKD, with a steeper decline in those with CKD. Higher baseline ePTFp and serum FGF23 were inversely correlated with eGFR slope. In multiple adjusted models, elevated ePTFp remained independently associated with faster eGFR decline, whereas the serum FGF23 association was attenuated after covariate adjustment.

**Conclusions:**

Elevated ePTFp was independently linked to accelerated kidney function decline, underscoring the clinical relevance of calcium phosphate microcrystallopathy. ePTFp may represent a practical biomarker with implications for prevention and treatment strategies targeting the aging kidney with proximal tubular phosphate exposure.

## GRAPHICAL ABSTRACT

**GRAPHICAL ABSTRACT:**
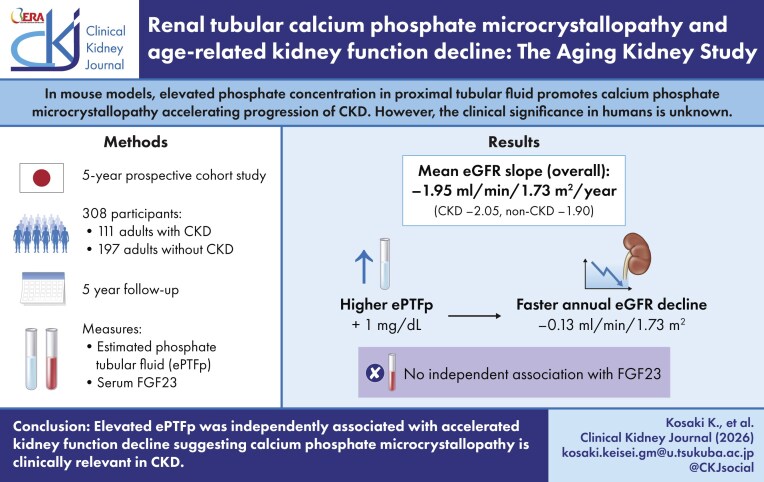

KEY LEARNING POINTS **What was known:**

- Phosphate burden has long been implicated in chronic kidney disease (CKD) progression.
- Basic studies propose that elevated phosphate in proximal tubular fluid triggers calcium phosphate formation, leading to tubular injury and nephron loss (i.e. microcrystallopathy).
- Clinical management, however, has mainly relied on elevated serum phosphate levels as a treatment trigger, which do not reflect tubular phosphate exposure.

**This study adds:**

- This study introduces estimated phosphate concentration in proximal tubular fluid (ePTFp) as a novel, noninvasive index of the aging kidney, estimating proximal tubular phosphate concentration.
- Higher ePTFp was independently associated with accelerated estimated glomerular filtration rate (eGFR) decline over 5 years.
- In contrast, the association between serum fibroblast growth factor 23 and eGFR slope was attenuated after full adjustment, underscoring the specificity of ePTFp as a risk marker.

**Potential impact:**

- ePTFp may serve as a practical biomarker for risk stratification in CKD progression and could help identify high-risk individuals for close monitoring and targeted interventions.
- In non-CKD populations, elevated ePTFp may act as an early warning marker of the aging kidney, informing preventive strategies before substantial nephron loss occurs.
- Our findings provide clinical support for the calcium phosphate microcrystallopathy model and may inform dietary and therapeutic approaches aimed at reducing phosphate exposure.

## INTRODUCTION

Chronic kidney disease (CKD) is a non-communicable condition defined by persistent kidney dysfunction and/or structural damage lasting ≥3 months [1]. As of 2021, an estimated 8.5% of the global population (approximately 674 million individuals) were affected by the disease, with prevalence projected to rise, particularly in developed nations with rapidly aging populations [2]. CKD develops through the interaction of primary underlying conditions such as diabetes mellitus and arterial hypertension, and an age-related decline in nephron number—the kidney’s functional units [3, 4]. Therefore, in addition to controlling established risk factors such as hypertension and hyperglycemia, it is essential to address common pathogenic mechanisms driving CKD progression across diverse etiologies.

The calcium phosphate microcrystallopathy model has recently been proposed as a novel pathogenic mechanism of CKD progression [5, 6]. According to this framework, when phosphate load exceeds the capacity of functioning nephrons, the excretory burden per nephron increases, elevating phosphate concentrations in the proximal tubular lumen. Once these concentrations surpass a critical threshold, calcium phosphate microcrystals form in the proximal tubular fluid. These microcrystals bind to toll-like receptor 4 (TLR-4) on the brush border of proximal tubular cells and are subsequently internalized via endocytosis. This process activates pro-inflammatory and pro-fibrotic signaling pathways within renal tissues. The resulting tubular injury drives persistent tubulointerstitial inflammation and fibrosis, establishing a self-perpetuating cycle of nephron loss and further deterioration of renal function.

According to the renal tubule luminal calcium phosphate microcrystallopathy model, elevated phosphate concentrations in the proximal tubular lumen, driven by increased phosphate excretion per nephron, may contribute to the pathogenesis of CKD progression. However, direct measurement of phosphate levels in proximal tubular fluid is technically and ethically infeasible in humans with current methodologies. Recently, a novel noninvasive method was developed to estimate phosphate concentration in the proximal tubular fluid near the S3 segment, derived from blood and urine samples under specific assumptions (see Materials and methods) [7]. In mouse models, the association between estimated phosphate concentration in proximal tubular fluid (ePTFp) and mRNA expression of markers of tubular injury, inflammation, and fibrosis followed a two-segment linear regression with an initial slope of zero [6]. These findings suggest that ePTFp could serve as a practical biomarker for calcium phosphate microcrystallopathy, a novel mechanistic pathway implicated in CKD progression.

We recently investigated whether the association between ePTFp and tubular injury markers observed in mouse models could also be identified in middle-aged and older adults with CKD stages G2–G4 [8]. When ePTFp was plotted on the x-axis against urinary liver-type fatty acid-binding protein (L-FABP) and β2-microglobulin on the y-axis, the association mirrored the two-segment linear regression pattern previously reported in mice [6]. These results suggest that higher ePTFp levels may reflect progressive tubulointerstitial injury driven by calcium phosphate microcrystallopathy, which, if unaddressed, could exacerbate aging-related CKD, distinct from traditional arteriosclerosis-related nephrosclerosis. Clinical evidence in humans, however, remains limited. To our knowledge, ePTFp was originally validated in experimental animal models and has been applied in humans only in our cross-sectional studies; longitudinal investigations evaluating the long-term predictive ability of ePTFp are lacking. Therefore, clarifying whether ePTFp contributes to long-term kidney function trajectories would provide essential clinical validation for the calcium phosphate microcrystallopathy model.

Epidemiological studies in clinical nephrology have established the longitudinal change in kidney function, expressed as the estimated glomerular filtration rate (eGFR) slope, as a reliable surrogate endpoint for CKD progression [9, 10]. Accordingly, this 5-year prospective cohort study aimed to examine whether ePTFp is independently associated with eGFR slope in adults with and without CKD. We hypothesized that higher baseline ePTFp would predict a steeper decline in kidney function over time.

## MATERIALS AND METHODS

### Study design and setting

The Aging Kidney Study was a single-center prospective observational cohort study conducted at the University of Tsukuba (UMIN000034741) to compare age-related changes in phosphate metabolism between adults from the general population and patients with CKD. Baseline assessments were performed during the 2018 academic year, with annual follow-up examinations conducted during the same period each year. Over 5 years of continuous follow-up, longitudinal data were collected at six time points. The study was approved by the Ethical Committee of the University of Tsukuba Hospital (approval no. H30-161) and conducted in accordance with the Declaration of Helsinki. All participants received a detailed explanation of study objectives and procedures before baseline assessments, and subsequently provided written informed consent.

### Study participants

**Figure 1:**
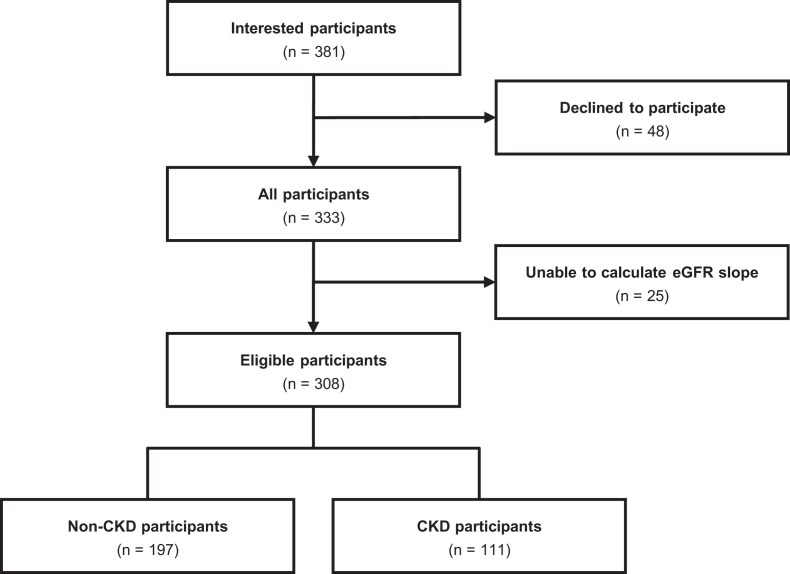
Study flow diagram. Participants with fewer than two eGFR measurements were excluded because the eGFR slope could not be calculated. Participants were stratified into non-CKD and CKD groups based on baseline eGFR <60 mL/min/1.73 m² and/or urinary ACR ≥30 mg/g.

Participants were recruited through community magazine advertisements, flyers, website postings targeting the southern Ibaraki Prefecture, and referrals from the Department of Nephrology at the University of Tsukuba Hospital. Between September 2018 and February 2019, 381 individuals applied; 48 declined for personal reasons after receiving detailed study information. Among the 333 participants who completed baseline examinations, 25 with fewer than two eGFR measurements were excluded because the primary outcome (eGFR slope) could not be calculated. The final cohort comprised 308 participants: 197 adults without CKD and 111 patients with CKD. CKD was defined according to established criteria as eGFR <60 mL/min/1.73 m² and/or urinary albumin-to-creatinine ratio (ACR) ≥30 mg/g [11]. The study flow diagram is presented in Fig. 1.

### eGFR slope

Serum creatinine and cystatin C concentrations were measured from early morning venous blood samples, and eGFR was subsequently calculated. eGFR based on serum creatinine (eGFR_cr_) and cystatin C (eGFR_cys_) were derived using standardized equations validated for the Japanese population [12, 13]. To improve accuracy, the mean values of eGFR_cr_ and eGFR_cys_ were computed and used for all subsequent analyses. The rate of eGFR change over time (eGFR slope) was estimated with a linear mixed-effects model [14]. In this model, elapsed time (years from baseline) was treated as a fixed effect, and participant-level variability as a random effect. The eGFR slope was expressed as mL/min/1.73 m²/year.

### ePTFp

The rationale for noninvasively estimating phosphate concentration in proximal tubular fluid near the S3 segment is outlined as follows. First, the phosphate concentration immediately after glomerular filtration (i.e. the phosphate concentration in glomerular filtrate) is assumed to be equal to that in blood. As the filtrate passes through the proximal tubule, phosphate is reabsorbed [15], and hence the concentration at the S3 segment equals serum phosphate multiplied by the fractional excretion of phosphate (FEp). Because approximately 70% of water is reabsorbed along the proximal tubule [16], solute concentrations increase about 3.33-fold by the time they reach the downstream segment. Thus, phosphate concentration in proximal tubular fluid can theoretically be estimated as: “serum phosphate × FEp × 3.33” [7]. Since FEp is the ratio of phosphate clearance (24-h urinary phosphate excretion divided by serum phosphate concentration) to creatinine clearance (24-h urinary creatinine excretion divided by serum creatinine concentration), the final formula for calculating ePTFp can be expressed as follows: “ePTFp ≡ serum phosphate × (urinary phosphate × 24-h urine volume/serum phosphate)/(urinary creatinine × 24-h urine volume/serum creatinine) × 3.33” = ”urinary phosphate/urinary creatinine × serum creatinine × 3.33” [7]. This estimation method has been validated by showing that the calculated ePTFp closely corresponds to phosphate concentration directly measured via micropuncture of the proximal tubular lumen in living rats [6, 17].

### Biochemical analysis

Blood samples were collected from the antecubital vein in the morning, centrifuged at 3000 rpm for 15 min at 4°C using a refrigerated centrifuge (AX-320, TOMY SEIKO Co., Ltd, Tokyo, Japan), and stored until biochemical analysis. Concentrations of high-density lipoprotein (HDL) cholesterol, low-density lipoprotein (LDL) cholesterol, triglycerides, glucose, and hemoglobin A1c (HbA1c) were determined at the Tsukuba i-Laboratory LLP (Tsukuba, Ibaraki, Japan) using standardized and validated methods. Serum fibroblast growth factor 23 (FGF23) concentrations were quantified with a sandwich enzyme-linked immunosorbent assay kit (Kainos Laboratories Inc., Tokyo, Japan) according to the manufacturer’s instructions [18]. Serum 1,25-dihydroxyvitamin D [1,25(OH)_2_D] and intact parathyroid hormone (int-PTH) concentrations were measured using radioimmunoassay (Immunodiagnostic Systems Holdings Ltd, Boldon, UK) and electrochemiluminescence immunoassay (Roche Diagnostics K.K., Tokyo, Japan) [19]. Urinary concentrations of creatinine, phosphate, albumin, and L-FABP were assessed from spot midstream urine samples collected concurrently with blood samples. Urinary albumin and L-FABP were standardized using urinary creatinine excretion ratios.

### Covariates

Body composition was assessed using a stadiometer (AD-6227R; A&D Inc., Tokyo, Japan) and a bioelectrical impedance analyzer (Inbody 770; Inbody Japan Inc., Tokyo, Japan). Body mass index (BMI) was calculated as weight in kilograms divided by height in meters squared. Obesity/overweight was defined as BMI ≥25 kg/m² [20]. Brachial blood pressure and heart rate (HR) were measured with a semi-automatic vascular assessment system (Form PWV/ABI, Colin Medical Technology, Aichi, Japan). Dietary phosphate intake was estimated using a validated self-administered food frequency questionnaire (FFQg ver.5; Kenpakusha Co., Ltd, Tokyo, Japan) [19]. Information on medication use and smoking status at baseline was obtained from personal medication records and a self-administered questionnaire. Hypertension, dyslipidemia, and diabetes were determined by integrating biochemical data with medication use. Hypertension was defined as systolic blood pressure (SBP) ≥140 mmHg, diastolic blood pressure (DBP) ≥90 mmHg, or use of antihypertensive medication. Dyslipidemia was defined as HDL cholesterol <40 mg/dL, LDL cholesterol ≥140 mg/dL, triglycerides ≥150 mg/dL, or use of lipid-lowering medication. Diabetes mellitus was defined as fasting blood glucose ≥126 mg/dL, HbA1c ≥6.5%, or use of glucose-lowering medication.

### Statistical analysis

Baseline characteristics were expressed as mean ± standard deviation, median with interquartile range, or frequency with percentage, as appropriate. eGFR values and both absolute and percentage changes from baseline during follow-up were reported as means with 95% confidence intervals (CIs). Baseline characteristics of adults without CKD and patients with CKD were compared using the Student’s *t*-test or Mann–Whitney U test for continuous variables and Fisher’s exact test for categorical variables. Simple correlations between the eGFR slope and ePTFp, serum FGF23, 1,25(OH)_2_D, and int-PTH levels were examined using Spearman’s rank correlation coefficient. For the primary analysis, multiple linear regression estimated regression coefficients and 95% CIs for the association of baseline ePTFp and serum FGF23 with eGFR slope during follow-up, adjusting for covariates. In hierarchical multiple linear regression models, adjustments included CKD status, interaction terms between CKD and ePTFp (ePTFp × CKD) or serum FGF23 (serum FGF23 × CKD), age, sex, obesity/overweight, hypertension, dyslipidemia, diabetes mellitus, current smoking status, eGFR, urinary ACR, urinary L-FABP, and dietary phosphate intake. Because dietary phosphate intake measures contained missing values, models including this variable were performed separately in participants with complete dietary data (*n* = 299). In addition, stratified regression analyses were performed separately for participants with and without CKD. Within each stratum, multiple linear regression models were constructed using the same hierarchical adjustment strategy as in the primary analysis, excluding the interaction terms, to estimate the association between baseline ePTFp and eGFR slope. Statistical analyses were conducted using SPSS Statistics, version 30.0 (IBM Inc., Armonk, NY, USA) and Stata, version 16.0 (Stata Corp., College Station, TX, USA). Statistical significance was defined *a priori* as *P* < .05.

## RESULTS

Baseline characteristics of the overall, non-CKD, and CKD groups are summarized in Table 1. In all participants, the mean age was 62 ± 12 years, 63% were female, and the mean eGFR was 80 ± 23 mL/min/1.73 m². Compared with adults without CKD, participants with CKD were older and had higher BMI, SBP, DBP, HR, triglyceride levels, fasting blood glucose levels, HbA1c levels, and higher prevalence of obesity/overweight, hypertension, and diabetes mellitus, as well as higher serum concentrations of creatinine, cystatin C, urinary ACR, urinary L-FABP, ePTFp, serum FGF23 and int-PTH. In contrast, participants with CKD had a lower proportion of females, and lower HDL and LDL cholesterol levels, eGFR, dietary phosphate intake, and serum 1,25(OH)₂D levels than did those without CKD. No significant differences were observed between groups for dyslipidemia prevalence, current smoking, serum phosphate concentrations, or urinary phosphate levels.

**Table 1:** Baseline characteristics of all, non-CKD, and CKD groups.

Variable	All (*n* = 308)	Non-CKD (*n* = 197)	CKD (*n* = 111)	*P*-value
Age, years	62 ± 12	61 ± 11	64 ± 11	.009
Women, *n* (%)	195 (63)	142 (72)	53 (48)	<.001
BMI, kg/m^2^	22.4 ± 3.4	22.1 ± 3.1	23.0 ± 3.9	.027
SBP, mmHg	121 ± 15	118 ± 14	127 ± 14	<.001
DBP, mmHg	73 ± 10	71 ± 10	77 ± 10	<.001
HR, bpm	61 ± 8	59 ± 8	63 ± 9	.001
HDL cholesterol, mg/dL	70 ± 18	73 ± 19	64 ± 17	<.001
LDL cholesterol, mg/dL	124 ± 29	130 ± 29	112 ± 27	<.001
Triglyceride, mg/dL	101 ± 65	88 ± 47	123 ± 85	<.001
Fasting blood glucose, mg/dL	103 ± 16	101 ± 15	106 ± 17	.004
HbA1c, %	5.8 ± 0.5	5.7 ± 0.4	5.9 ± 0.6	.007
Overweight/obesity, *n* (%)	62 (20)	31 (16)	31 (28)	.010
Hypertension, *n* (%)	116 (38)	37 (19)	79 (71)	<.001
Dyslipidemia, *n* (%)	159 (52)	94 (48)	65 (59)	.068
Diabetes mellitus, *n* (%)	35 (11)	12 (6)	23 (21)	<.001
Current smoking status, *n* (%)	6 (2)	3 (2)	3 (3)	.472
Serum creatinine, mg/dL	0.83 ± 0.41	0.66 ± 0.14	1.13 ± 0.53	<.001
Serum cystatin C, mg/dL	0.90 ± 0.36	0.72 ± 0.11	1.20 ± 0.45	<.001
eGFR, mL/min/1.73m^2^	80 ± 23	91 ± 15	59 ± 22	<.001
Urinary ACR, mg/g	14 (7–38)	9 (6–14)	74 (35–372)	<.001
Urinary L-FABP, μg/g.Cr	1.3 (0.5–3.1)	0.9 (0.4–1.8)	2.8 (1.2–6.2)	<.001
Serum phosphate, mg/dL	3.46 ± 0.45	3.49 ± 0.43	3.39 ± 0.48	.056
Urinary phosphate, mg/dL	35.9 ± 24.7	37.2 ± 27.4	33.5 ± 19.0	.166
ePTFp, mg/dL	1.38 ± 0.69	1.15 ± 0.36	1.78 ± 0.92	<.001
Dietary phosphate intake, mg/day^a^	1076 ± 300	1118 ± 297	997 ± 291	<.001
Serum FGF23, pg/mL	46 (35–62)	42 (32–52)	62 (44–83)	<.001
Serum 1,25(OH)_2_D, pg/mL	62.2 ± 18.2	65.8 ± 17.2	55.8 ± 18.3	<.001
Serum int-PTH, pg/mL	40 (33–50)	38 (33–47)	44 (35–60)	<.001

Values are presented as the means ± standard deviation, the median (interquartile range) or frequency counts (%).

a Data available in 299 individuals.

Mean values with 95% CIs for eGFR, as well as absolute and percentage changes from baseline during follow-up, are provided in Supplemental Table S1. Across the 5-year follow-up, eGFR declined gradually regardless of CKD status. Patients with CKD, who had lower baseline eGFR, experienced a greater decline than did participants without CKD. The mean eGFR slope was –1.95 ± 0.46 mL/min/1.73 m²/year in the overall cohort, –1.90 ± 0.44 mL/min/1.73 m²/year in participants without CKD, and –2.05 ± 0.48 mL/min/1.73 m²/year in patients with CKD.

**Figure 2:**
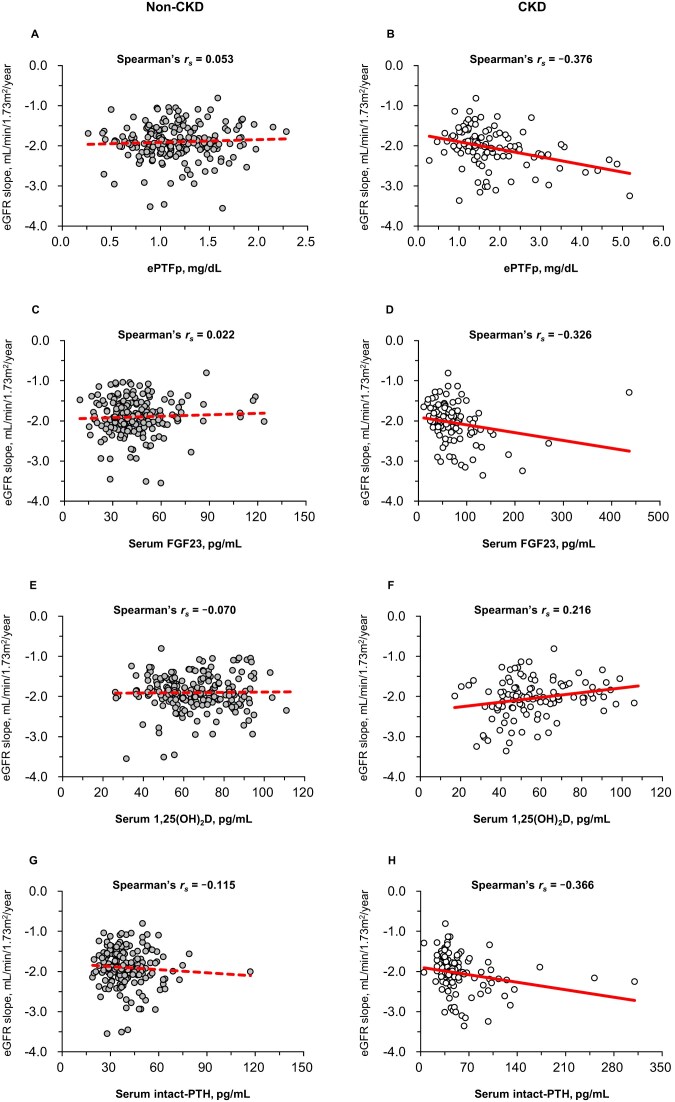
Scatter plots of the eGFR slope with ePTFp, serum FGF23, serum 1,25(OH)₂D, and serum int-PTH in participants with and without CKD..

Figure 2 presents the simple correlations between eGFR slope and ePTFp, serum FGF23, 1,25(OH)₂D, and int-PTH levels in participants without CKD and with CKD. In adults with CKD, negative correlations were observed between eGFR slope and ePTFp (Fig. 2B), serum FGF23 levels (Fig. 2D), and serum int-PTH levels (Fig. 2H). In contrast, a positive correlation was identified between eGFR slope and serum 1,25(OH)₂D levels (Fig. 2F). Among adults without CKD, no significant correlations were observed between eGFR slope and ePTFp (Fig. 2A), serum FGF23 (Fig. 2C), serum 1,25(OH)₂D (Fig. 2E), or serum int-PTH (Fig. 2G) levels.

Table 2 presents regression coefficients and 95% CIs for the association between baseline ePTFp and eGFR slope during follow-up. In Model 1, which included CKD status and the interaction between CKD and ePTFp, baseline ePTFp was negatively associated with eGFR slope (B = –0.189; 95% CI –0.280 to –0.098). This association remained statistically significant after adjustment for covariates, including age, sex, obesity/overweight, hypertension, dyslipidemia, diabetes mellitus, and current smoking status (Model 2), and persisted after further adjustment for baseline eGFR, urinary ACR, and urinary L-FABP levels (Model 3). Moreover, in Supplemental Table S2, further adjustment for estimated dietary phosphate intake (*n* = 299) did not materially alter the association, and baseline ePTFp remained negatively associated with eGFR slope (B = –0.124; 95% CI –0.240 to –0.009).

**Table 2:** Regression coefficients and 95% CIs for the association between baseline ePTFp and eGFR slope at follow-up.

	Model 1			Model 2			Model 3		
All (*n* = 308)	B (95%CI)	*β*	*P*-value	B (95%CI)	*β*	*P*-value	B (95%CI)	*β*	*P*-value
ePTFp, mg/dL	–0.189 (–0.280, –0.098)	–0.28	<.001	–0.165 (–0.256, –0.073)	–0.25	<.001	–0.133 (–0.244, –0.022)	–0.20	.019
CKD (no)	–0.271 (–0.550, 0.007)	–0.28	.056	–0.283 (–0.571, 0.005)	–0.29	.054	–0.348 (–0.649, –0.048)	–0.36	.023
ePTFp × CKD	0.257 (0.060, 0.454)	0.35	.011	0.217 (0.019, 0.416)	0.29	.032	0.187 (–0.016, 0.389)	0.25	.070
Age, years				0.002 (–0.003, 0.006)	0.04	.478	0.002 (–0.003, 0.006)	0.04	.467
Sex (women)				0.011 (–0.099, 0.120)	0.01	.850	0.033 (–0.079, 0.146)	0.03	.560
Overweight/obesity (yes)				–0.065 (–0.197, 0.067)	–0.06	.332	–0.066 (–0.197, 0.066)	–0.06	.329
Hypertension (yes)				–0.113 (–0.239, 0.013)	–0.12	.079	–0.095 (–0.225, 0.034)	–0.10	.147
Dyslipidemia (yes)				–0.060 (–0.164, 0.044)	–0.07	.255	–0.040 (–0.148, 0.069)	–0.04	.470
Diabetes mellitus (yes)				0.023 (–0.139, 0.185)	.02	0.784	0.029 (–0.134, 0.191)	0.02	.729
Current smoking status (yes)				–0.477 (–0.840, –0.115)	–.14	0.010	–0.416 (–0.782, –0.050)	–0.12	.026
eGFR, mL/min/1.73 m^2^							–0.0004 (–0.004, 0.003)	–0.02	.838
Urinary ACR, mg/g^a^							–0.121 (–0.247, 0.006)	–0.18	.061
Urinary L-FABP, μg/g.Cr^a^							–0.023 (–0.124, 0.078)	–0.03	.660

B and *β* indicate unstandardized and standardized regression coefficients, respectively.

a Log transformed.

The results of the stratified regression analyses are presented in Supplemental Tables S3 and S4. In non-CKD participants, baseline ePTFp was not significantly associated with eGFR slope in any of the models. In contrast, among participants with CKD, baseline ePTFp was significantly associated with eGFR slope (Models 1 and 2); however, this association was attenuated after further adjustment for baseline eGFR, urinary ACR, and urinary L-FABP levels (Model 3).

Table 3 presents regression coefficients and 95% CIs for the association between baseline serum FGF23 levels and eGFR slope during follow-up. In Model 1, which included CKD status and the interaction between CKD and serum FGF23 levels, serum FGF23 levels were negatively associated with eGFR slope (B = –0.002; 95% CI –0.004 to –0.00003). This association was no longer significant after adjustment for additional confounders in Models 2 and 3.

**Table 3:** Regression coefficients and 95% CIs for the association between baseline serum FGF23 and eGFR slope at follow-up.

	Model 1			Model 2			Model 3		
All (*n* = 308)	B (95%CI)	*β*	*P*-value	B (95%CI)	*β*	*P*-value	B (95%CI)	*β*	*P*-value
Serum FGF23, pg/mL	–0.002 (–0.004, 0.000)	–0.16	.018	–0.001 (–0.002, 0.001)	–0.07	.237	0.000 (–0.002, 0.001)	–0.03	.615
CKD (no)	–0.050 (–0.270, 0.171)	–0.05	.657	0.042 (–0.087, 0.170)	0.04	.523	–0.135 (–0.312, 0.043)	–0.14	.136
Serum FGF23 × CKD	0.003 (–0.001, 0.007)	0.18	.109						
Age, years				0.002 (–0.002, 0.007)	0.06	.318	0.003 (–0.002, 0.008)	0.07	.229
Sex (women)				0.013 (–0.099, 0.125)	0.01	.814	0.033 (–0.081, 0.147)	0.03	.571
Overweight/obesity (yes)				–0.070 (–0.204, 0.064)	–0.06	.302	–0.070 (–0.202, 0.062)	–0.06	.298
Hypertension (yes)				–0.132 (–0.260, –0.004)	–0.14	.043	–0.096 (–0.226, 0.035)	–0.10	.150
Dyslipidemia (yes)				–0.058 (–0.164, 0.047)	–0.06	.279	–0.025 (–0.133, 0.083)	–0.03	.654
Diabetes mellitus (yes)				0.056 (–0.109, 0.221)	0.04	.502	0.043 (–0.122, 0.208)	0.03	.606
Current smoking status (yes)				–0.484 (–0.852, –0.115)	–0.15	.010	–0.394 (–0.763, –0.026)	–0.12	.036
eGFR, mL/min/1.73 m^2^							0.001 (–0.003, 0.005)	0.05	.571
Urinary ACR, mg/g^a^							–0.143 (–0.269, –0.017)	–0.21	.026
Urinary L-FABP, μg/g.Cr^a^							–0.049 (–0.149, 0.051)	–0.06	.335

B and *β* indicate unstandardized and standardized regression coefficients, respectively.

a Log transformed.

## DISCUSSION

This study aimed to examine whether baseline ePTFp is independently associated with longitudinal changes in eGFR (i.e. eGFR slope) among adults with and without CKD. The main findings were as follows. First, patients with CKD, who had lower baseline eGFR, showed a significantly greater annual decline in eGFR than did participants without CKD. Second, baseline ePTFp was negatively associated with eGFR slope, and this association remained after adjustment for multiple confounders, including baseline eGFR, urinary ACR, and urinary L-FABP. Third, although baseline serum FGF23 initially demonstrated a negative association with eGFR slope, this association was attenuated after adjustment for additional confounders. Overall, elevated ePTFp may serve as a biomarker for identifying individuals at increased risk of accelerated CKD progression due to renal tubular calcium phosphate microcrystallopathy.

**Figure 3:**
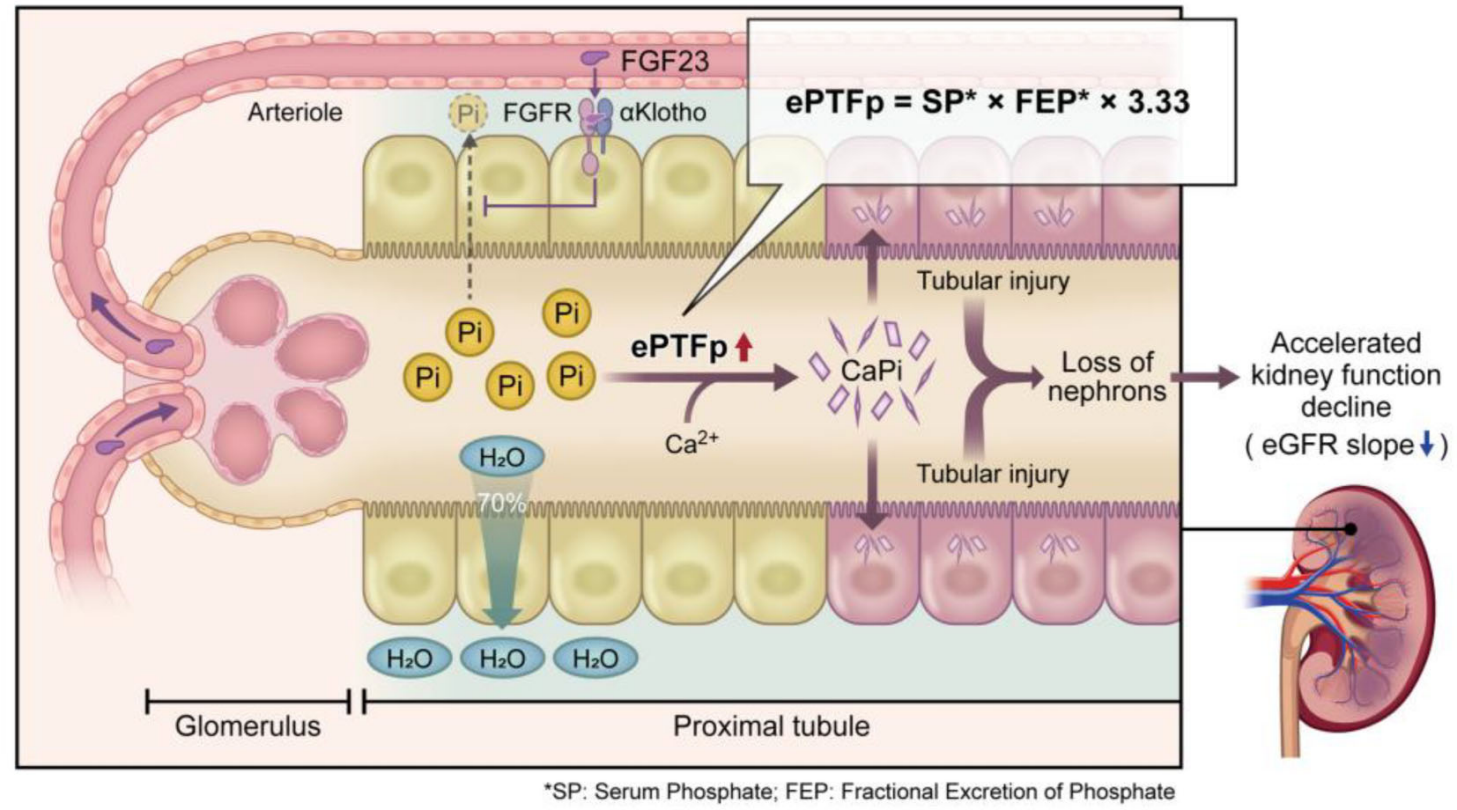
Proposed mechanistic pathway linking phosphate burden, renal tubular calcium phosphate microcrystallopathy, and kidney function decline. Schematic illustration of the proposed pathway by which increased phosphate burden elevates proximal tubular phosphate concentration, represented by ePTFp. When intraluminal phosphate exceeds a pathogenic threshold, calcium phosphate (CaPi) microcrystals form and induce tubular injury through inflammatory and fibrotic signaling. This process promotes tubular injury and nephron loss, contributing to kidney function decline. Clinically, ePTFp serves as a noninvasive marker of proximal tubular phosphate exposure and is associated with longitudinal eGFR slope. FGFR, fibroblast growth factor receptor.

Experimental evidence supporting phosphate burden–induced nephrotoxicity has been recognized for decades [21, 22]. Sustained phosphate loading causes tubular injury within weeks and overt interstitial fibrosis within months [23]. However, the underlying pathophysiological mechanisms were not fully understood. More recently, Shiizaki and colleagues demonstrated in animal models that elevated phosphate concentration in proximal tubular fluid promotes calcium phosphate crystal formation, directly injuring tubular cells through TLR4-mediated inflammation and fibrosis [6]. Moe further emphasized this crystallopathic mechanism as a self-perpetuating pathway that accelerates nephron loss and represents a potential therapeutic target in clinical practice [24]. Consistent with these experimental data, our clinical findings showed that higher baseline ePTFp, a marker of proximal tubular phosphate exposure, was independently associated with accelerated kidney function decline over 5 years (Fig. 3). Therefore, this investigation extends prior experimental evidence by applying ePTFp and demonstrating its independent association with long-term eGFR decline.

Beyond the present clinical findings, prior studies have shown that greater urinary phosphate excretion is associated with CKD progression in patients with CKD stages G2–G3 [25]. Furthermore, a higher ratio of urinary phosphate excretion to creatinine clearance has been linked to CKD progression within 3 years [26]. Importantly, these associations have been reported even in settings where serum phosphate levels remain within the normal range, underscoring the limited sensitivity of serum phosphate as a marker for detecting early phosphate-related kidney damage. Collectively, these observations support the translational relevance of the calcium phosphate microcrystallopathy model and suggest that ePTFp could function as a practical biomarker in clinical settings. This concept is particularly relevant in early CKD and the aging kidney, conditions in which systemic phosphate homeostasis is often preserved despite substantial increases in phosphate burden at the tubular level. Monitoring ePTFp in at-risk populations may facilitate early identification of individuals susceptible to accelerated CKD progression, allowing timely initiation of therapeutic strategies to reduce tubular phosphate exposure and potentially prevent irreversible nephron loss.

In hierarchical multiple linear regression models, ePTFp remained significantly associated with eGFR slope after full adjustment (Table 2), whereas the association for serum FGF23 was attenuated (Table 3). This difference likely reflects the fact that ePTFp directly estimates phosphate concentration within proximal tubular fluid—the site of crystallopathic injury—and therefore captures pathogenic phosphate exposure even when serum phosphate concentrations remain within the normal range. In contrast, serum FGF23 represents a systemic regulator influenced by multiple factors beyond tubular phosphate exposure [27, 28]. ePTFp may more precisely capture whether luminal phosphate has exceeded a harmful threshold, while serum FGF23 provides a broader, less specific signal that is diminished after adjustment for kidney function and other covariates. Consequently, ePTFp may better reflect the local pathogenic environment and serve as a more specific marker of risk for phosphate-induced tubular injury, whereas serum FGF23 remains informative as an indicator of overall mineral metabolism stress.

However, the estimation of ePTFp relies on several physiological assumptions, including an average proximal tubular water reabsorption of approximately 70%, corresponding to a 3.33-fold concentration of solutes along the proximal tubule [16]. In reality, proximal tubular water handling may vary across individuals and disease stages, particularly in CKD, where tubular function can be altered by aging, nephron loss, or adaptive responses. Deviations from the assumed 3.33 concentration factor could influence the absolute value of ePTFp; however, such variability would be expected to introduce non-differential measurement error, which would likely bias associations toward the null rather than generate spurious correlations. Therefore, the observed association between ePTFp and eGFR slope is likely to be conservative. Importantly, ePTFp is intended to function as an integrated index of proximal tubular phosphate exposure rather than a precise quantitative measurement of intraluminal phosphate concentration. From this perspective, its clinical utility is in risk stratification rather than exact physiological quantification.

Furthermore, unlike conventional biomarkers, ePTFp is conceptually distinct in that it estimates phosphate concentration within proximal tubular fluid, the anatomical compartment where renal tubular calcium phosphate microcrystallopathy is proposed to occur. This feature positions ePTFp closer to the putative site of injury than systemic markers such as serum phosphate or endocrine regulators such as serum FGF23. Consequently, ePTFp may function as a biomarker linking age-related nephron loss—the fundamental substrate of CKD progression—to downstream tubulointerstitial injury mediated by phosphate overload. In this context, ePTFp has the potential to capture CKD progression driven by diverse sources of phosphate burden, including both endogenous and exogenous factors. Therefore, ePTFp is not intended to replace established biomarkers of CKD progression, but rather to complement them by identifying individuals in whom phosphate-driven tubular injury contributes disproportionately to disease progression. This distinction may improve risk stratification and support the development of personalized preventive strategies targeting phosphate burden, particularly in aging populations.

It is also possible that lower baseline eGFR or subclinical glomerular or tubular damage contributes to elevated ePTFp. In this study, baseline eGFR, urinary ACR, and urinary L-FABP were included in multivariable models (Model 3 of Table 2), and the association between ePTFp and eGFR slope remained evident. This result suggests that ePTFp is not solely a surrogate of pre-existing glomerular or tubular damage. Nevertheless, given the close biological interplay between nephron loss, glomerular or tubular damage, and phosphate handling, partial mediation cannot be excluded. Mechanistically, ePTFp may reflect a dynamic process in which early tubular damage and altered phosphate handling reinforce each other, ultimately contributing to progressive tubulointerstitial injury. Future studies incorporating formal mediation analyses and longitudinal assessment of tubular injury biomarkers will be required to disentangle these pathways more definitively.

The significant interaction between ePTFp and CKD status (ePTFp × CKD) suggests that disease stage modifies the association between ePTFp and eGFR slope. In patients with CKD, reduced nephron number diminishes nephron reserve and increases phosphate load per remaining nephron, thereby increasing the likelihood that a given level of proximal tubular phosphate exposure exceeds a pathogenic threshold that induces calcium phosphate crystallization, tubular damage, and accelerated nephron loss. In contrast, most adults without CKD retain sufficient nephron reserve and buffering capacity [3, 29], allowing similar absolute ePTFp values to remain below this injury threshold. As a result, similar ePTFp values may not initiate the self-perpetuating cycle of nephron loss and progressive decline in renal function. This nephron number–dependent, threshold-based mechanism provides a biologically plausible explanation for the weaker association between ePTFp and eGFR slope observed in non-CKD participants. These findings underscore ePTFp as a potential tool for risk stratification in eGFR decline—identifying patients already at high risk, while serving in non-CKD populations as an early warning marker to guide preventive interventions before substantial functional loss occurs.

To our knowledge, this is the first study to evaluate the association between ePTFp—a physiologically grounded index of proximal tubular phosphate exposure—and long-term eGFR decline in a prospective cohort of both CKD and non-CKD participants. Its strengths include a 5-year follow-up with repeated eGFR measurements, allowing robust estimation of eGFR slope, a validated surrogate endpoint for CKD progression. The primary analysis also adjusted for an extensive set of potential confounders, including baseline kidney function, glomerular and tubular injury markers, enabling assessment of the independent association of ePTFp with eGFR slope. However, this study has several limitations. First, ePTFp was calculated from a single baseline set of blood and urine samples, which may not reflect intra-individual variability over time. Such variability could result from changes in diet, hydration status, medication use, or tubular phosphate handling. In addition, the temporal stability of ePTFp has not been systematically evaluated in longitudinal human studies, and it remains unclear how ePTFp changes with aging or CKD progression. From an epidemiological perspective, reliance on a single baseline measurement may introduce regression dilution bias, likely attenuating observed associations. Consequently, the association between baseline ePTFp and subsequent eGFR slope observed in this study may represent a conservative estimate of the true relationship between proximal tubular phosphate exposure and kidney function decline. Future studies incorporating repeated ePTFp measurements are needed to better characterize its temporal stability and variability, and refine risk prediction. Second, although ePTFp is designed to approximate proximal tubular phosphate concentration, it is an indirect index based on physiological assumptions, as direct measurement in humans is not currently feasible. Third, the cohort comprised Japanese adults with mostly normal kidney function or moderate CKD, which may limit generalizability. The Japanese dietary patterns, characterized by relatively lower intake of processed foods and inorganic phosphate additives, along with population-specific genetic and physiological differences in phosphate handling, may influence the relationship between phosphate burden and kidney injury. Therefore, caution is warranted when extrapolating these findings to Western or multi-ethnic populations with different dietary exposures, nephron reserve, or CKD etiologies. Fourth, although analyses accounted for multiple confounders, residual confounding cannot be excluded. In particular, dietary phosphate intake—an important determinant of phosphaturia and fractional phosphate excretion—may confound the interpretation of ePTFp. Although dietary phosphate intake estimated using a self-administered food frequency questionnaire was additionally addressed in a separate regression model (Supplemental Table S2), objective biological assessment based on urinary excretion (i.e. 24-h urine collections) was not performed in this study. This lack of direct measurement represents a key limitation and may limit the ability to fully separate proximal tubular phosphate exposure from nutritional effects. Finally, the observational design precludes causal inference; interventional studies are required to determine whether lowering ePTFp mitigates CKD progression and improves kidney outcomes.

In this 5-year prospective cohort study, higher baseline ePTFp—a surrogate marker of proximal tubular phosphate exposure—was independently associated with accelerated kidney function decline, particularly in patients with CKD. These findings support the calcium phosphate microcrystallopathy model and underscore the potential of ePTFp as a biomarker for risk stratification. Monitoring proximal tubular phosphate exposure may aid in the early detection of CKD progression and help prevent nephron loss associated with aging. Future interventional studies are needed to determine whether lowering ePTFp mitigates tubular injury and slows CKD progression.

## Supplementary Material

sfag085_Supplemental_File

## Data Availability

The datasets generated and analyzed during the current study are not publicly available due to ethical restrictions. According to the Ethics Committee of the University of Tsukuba Hospital (approval no. H30-161), individual-level clinical and laboratory data contain sensitive personal information and cannot be shared outside the institution to protect the privacy of participants. Therefore, no accession numbers or external URLs are provided. Researchers who are interested in accessing specific analyses related to this dataset may contact the corresponding author. Subject to approval by the Ethics Committee of the University of Tsukuba, de-identified summary data or additional analyses performed by the study team may be made available upon reasonable request.
