# Supplementary material for: Renal tubular calcium phosphate microcrystallopathy and age-related kidney function decline: The Aging Kidney Study
Source: Clin Kidney J. 2026 Mar 16;19(5):sfag085. doi: 10.1093/ckj/sfag085 (PMC13133628; doi:10.1093/ckj/sfag085)
Supplement: sfag085_Supplemental_File [file sfag085_supplemental_file.docx]

**Supplemental Table 1.** Mean and 95% confidence interval for eGFR over the follow-up period

|  | **Baseline**  **(2018)** | |  | **1 year**  **(2019)** | |  | **2 year**  **(2020)** | |  | **3 year**  **(2021)** | |  | **4 year**  **(2022)** | |  | **5 year**  **(2023)** | |
| --- | --- | --- | --- | --- | --- | --- | --- | --- | --- | --- | --- | --- | --- | --- | --- | --- | --- |
| **n** |  |  |  |  |  |  |  |  |  |  |  |  |  |  |  |  |  |
| All | 308 | |  | 304 | |  | 72 | |  | 203 | |  | 219 | |  | 217 | |
| Non-CKD | 197 | |  | 197 | |  | 51 | |  | 140 | |  | 154 | |  | 155 | |
| CKD | 111 | |  | 107 | |  | 21 | |  | 63 | |  | 65 | |  | 62 | |
| **eGFR, mL/min/1.73m^2^** | | | | | | | | | | | | | | | | | |
| All | 79.6 | (77.0, 82.3) |  | 76.9 | (74.3, 79.5) |  | 80.0 | (74.1, 85.8) |  | 76.8 | (73.7, 79.9) |  | 76.2 | (73.2, 79.2) |  | 72.5 | (69.7, 75.2) |
| Non-CKD | 91.2 | (89.1, 93.3) |  | 87.6 | (85.6, 89.7) |  | 89.1 | (85.0, 93.1) |  | 85.6 | (83.2, 87.9) |  | 85.3 | (82.8, 87.8) |  | 80.3 | (78.1, 82.5) |
| CKD | 59.2 | (55.1, 63.3) |  | 57.1 | (52.8, 61.4) |  | 57.9 | (43.8, 72.0) |  | 57.3 | (51.0, 63.7) |  | 54.7 | (49.3, 60.0) |  | 52.9 | (47.4, 58.4) |
| **Change from baseline in eGFR, mL/min/1.73m^2^** | | | | | | | | | | | | | | | | | |
| All | - | |  | -3.06 | (-3.65, -2.47) |  | -2.62 | (-4.01, -1.23) |  | -5.01 | (-5.93, -4.09) |  | -6.38 | (-7.31, -5.44) |  | -11.12 | (-11.96, -10.28) |
| Non-CKD | - | |  | -3.56 | (-4.31, -2.81) |  | -2.34 | (-3.95, -0.73) |  | -5.36 | (-6.43, -4.29) |  | -6.31 | (-7.41, -5.21) |  | -11.19 | (-12.18, -10.21) |
| CKD | - | |  | -2.15 | (-3.10, -1.20) |  | -3.29 | (-6.23, -0.35) |  | -4.23 | (-6.05, -2.41) |  | -6.53 | (-8.35, -4.72) |  | -10.93 | (-12.56, -9.29) |
| **%Change from baseline in eGFR, %** | | | | | | | | | | | | | | | | | |
| All | - | |  | -4.06 | (-4.92, -3.19) |  | -4.83 | (-7.63, -2.03) |  | -7.21 | (-8.89, -5.53) |  | -8.80 | (-10.44, -7.17) |  | -14.25 | (-15.67, -12.83) |
| Non-CKD | - | |  | -3.81 | (-4.59, -3.02) |  | -2.42 | (-4.14, -0.69) |  | -5.79 | (-6.93, -4.66) |  | -7.04 | (-8.23, -5.85) |  | -12.21 | (-13.24, -11.17) |
| CKD | - | |  | -4.52 | (-6.53, -2.50) |  | -10.70 | (-19.33, -2.06) |  | -10.37 | (-15.16, -5.58) |  | -12.98 | (-17.65, -8.32) |  | -19.34 | (-23.40, -15.28) |

CKD, chronic kidney disease; eGFR, estimated glomerular filtration rate

**Supplemental Table 2.** Association between baseline ePTFp and eGFR slope after additional adjustment for dietary phosphate intake

| **All (n = 299)** | **B (95%CI)** | ***β*** | ***P*-value** |
| --- | --- | --- | --- |
| ePTFp, mg/dL | -0.124 (-0.240, -0.009) | -0.18 | 0.034 |
| CKD (no) | -0.333 (-0.648, -0.019) | -0.34 | 0.038 |
| ePTFp*CKD | 0.178 (-0.031, 0.388) | 0.24 | 0.095 |
| Age, yr | 0.002 (-0.003, 0.007) | 0.05 | 0.429 |
| Sex (women) | 0.028 (-0.088, 0.144) | 0.03 | 0.634 |
| Overweight/obesity (yes) | -0.068 (-0.204, 0.067) | -0.06 | 0.322 |
| Hypertension (yes) | -0.104 (-0.239, 0.031) | -0.11 | 0.129 |
| Dyslipidemia (yes) | -0.042 (-0.153, 0.070) | -0.04 | 0.463 |
| Diabetes mellitus (yes) | 0.024 (-0.142, 0.191) | 0.02 | 0.774 |
| Current smoking status (yes) | -0.426 (-0.799, -0.054) | -0.13 | 0.025 |
| eGFR, mL/min/1.73m^2^ | -0.0003 (-0.004, 0.004) | -0.01 | 0.889 |
| Urinary ACR, mg/g^a^ | -0.114 (-0.244, 0.017) | -0.17 | 0.087 |
| Urinary L-FABP, μg/g.Cr^a^ | -0.024 (-0.128, 0.080) | -0.03 | 0.654 |
| Dietary phosphate intake, mg/day | -0.00003 (-0.0002, 0.0002) | -0.02 | 0.783 |

B and *β* indicates unstandardized and standardized regression coefficients, respectively. ^a^Log transformed. ePTFp, estimated proximal tubule fluid phosphate concentration; CKD, chronic kidney disease; eGFR, estimated glomerular filtration rate; ACR, albumin-to-creatinine ratio; L-FABP, liver-type fatty acid-binding protein.

**Supplemental Table 3.** Stratified regression analysis of the association between baseline ePTFp and eGFR slope in participants without CKD

| **Non-CKD (n = 197)** | **Model 1** | | |  | **Model 2** | | |  | **Model 3** | | |
| --- | --- | --- | --- | --- | --- | --- | --- | --- | --- | --- | --- |
|  | **B (95%CI)** | ***β*** | ***P*-value** |  | **B (95%CI)** | ***β*** | ***P*-value** |  | **B (95%CI)** | ***β*** | ***P*-value** |
| ePTFp, mg/dL | 0.068 (-0.107, 0.243) | 0.05 | 0.446 |  | 0.047 (-0.132, 0.225) | 0.04 | 0.607 |  | 0.039 (-0.143, 0.222) | 0.03 | 0.670 |
| Age, yr |  |  |  |  | 0.002 (-0.004, 0.008) | 0.04 | 0.558 |  | 0.000 (-0.007, 0.008) | 0.01 | 0.917 |
| Sex (women) |  |  |  |  | -0.033 (-0.176, 0.109) | -0.03 | 0.644 |  | -0.065 (-0.219, 0.090) | -0.07 | 0.410 |
| Overweight/obesity (yes) |  |  |  |  | -0.123 (-0.305, 0.058) | -0.10 | 0.182 |  | -0.110 (-0.294, 0.073) | -0.09 | 0.238 |
| Hypertension (yes) |  |  |  |  | -0.077 (-0.249, 0.095) | -0.07 | 0.377 |  | -0.095 (-0.270, 0.079) | -0.08 | 0.281 |
| Dyslipidemia (yes) |  |  |  |  | -0.061 (-0.190, 0.068) | -0.07 | 0.352 |  | -0.081 (-0.215, 0.054) | -0.09 | 0.237 |
| Diabetes mellitus (yes) |  |  |  |  | -0.202 (-0.464, 0.060) | -0.11 | 0.131 |  | -0.213 (-0.482, 0.057) | -0.11 | 0.121 |
| Current smoking status (yes) |  |  |  |  | -0.471 (-0.980, 0.039) | -0.13 | 0.070 |  | -0.471 (-0.982, 0.039) | -0.13 | 0.070 |
| eGFR, mL/min/1.73m^2^ |  |  |  |  |  |  |  |  | -0.001 (-0.006, 0.004) | -0.03 | 0.741 |
| Urinary ACR, mg/g^a^ |  |  |  |  |  |  |  |  | 0.206 (-0.094, 0.507) | 0.12 | 0.177 |
| Urinary L-FABP, μg/g.Cr^a^ |  |  |  |  |  |  |  |  | -0.050 (-0.178, 0.077) | -0.06 | 0.438 |

B and *β* indicates unstandardized and standardized regression coefficients, respectively. ^a^Log transformed. ePTFp, estimated proximal tubule fluid phosphate concentration; CKD, chronic kidney disease; eGFR, estimated glomerular filtration rate; ACR, albumin-to-creatinine ratio; L-FABP, liver-type fatty acid-binding protein.

**Supplemental Table 4.** Stratified regression analysis of the association between baseline ePTFp and eGFR slope in participants with CKD

| **CKD (n = 111)** | **Model 1** | | |  | **Model 2** | | |  | **Model 3** | | |
| --- | --- | --- | --- | --- | --- | --- | --- | --- | --- | --- | --- |
|  | **B (95%CI)** | ***β*** | ***P*-value** |  | **B (95%CI)** | ***β*** | ***P*-value** |  | **B (95%CI)** | ***β*** | ***P*-value** |
| ePTFp, mg/dL | -0.189 (-0.281, -0.097) | -0.36 | < 0.001 |  | -0.151 (-0.246, -0.056) | -0.29 | 0.002 |  | -0.111 (-0.236, 0.014) | -0.21 | 0.082 |
| Age, yr |  |  |  |  | 0.002 (-0.006, 0.010) | 0.05 | 0.625 |  | 0.001 (-0.006, 0.009) | 0.03 | 0.763 |
| Sex (women) |  |  |  |  | 0.075 (-0.106, 0.256) | 0.08 | 0.415 |  | 0.098 (-0.089, 0.285) | 0.10 | 0.303 |
| Overweight/obesity (yes) |  |  |  |  | -0.026 (-0.230, 0.177) | -0.02 | 0.798 |  | -0.016 (-0.215, 0.183) | -0.02 | 0.873 |
| Hypertension (yes) |  |  |  |  | -0.154 (-0.354, 0.046) | -0.15 | 0.131 |  | -0.082 (-0.290, 0.125) | -0.08 | 0.433 |
| Dyslipidemia (yes) |  |  |  |  | -0.052 (-0.236, 0.131) | -0.05 | 0.573 |  | 0.009 (-0.183, 0.202) | 0.01 | 0.924 |
| Diabetes mellitus (yes) |  |  |  |  | 0.146 (-0.066, 0.359) | 0.12 | 0.176 |  | 0.156 (-0.052, 0.363) | 0.13 | 0.140 |
| Current smoking status (yes) |  |  |  |  | -0.475 (-1.019, 0.069) | -0.16 | 0.087 |  | -0.305 (-0.856, 0.247) | -0.10 | 0.276 |
| eGFR, mL/min/1.73m^2^ |  |  |  |  |  |  |  |  | 0.000 (-0.006, 0.006) | 0.00 | 0.992 |
| Urinary ACR, mg/g^a^ |  |  |  |  |  |  |  |  | -0.207 (-0.356, -0.059) | -0.30 | 0.007 |
| Urinary L-FABP, μg/g.Cr^a^ |  |  |  |  |  |  |  |  | 0.0000 (-0.177, 0.177) | 0.00 | 1.000 |

B and *β* indicates unstandardized and standardized regression coefficients, respectively. ^a^Log transformed. FGF23, fibroblast growth factor 23; CKD, chronic kidney disease; eGFR, estimated glomerular filtration rate; ACR, albumin-to-creatinine ratio; L-FABP, liver-type fatty acid-binding protein.
